# The effect of job aids on knowledge retention among Patent and Proprietary Medicine Vendors trained to administer injectable contraceptives: longitudinal results from implementation science in Nigeria

**DOI:** 10.1186/s12889-019-7668-2

**Published:** 2019-10-24

**Authors:** Sara Chace Dwyer, Aparna Jain, Salisu Mohammed Ishaku, Faizah Tosin Okunade, Chiamaka Uzomba, Adedamola Adebayo, Elizabeth Tobey

**Affiliations:** 1 Population Council, Washington, DC, 20008 USA; 2 Population Council, Abuja, Nigeria; 3 Department of Epidemiology and Medical Statistics, University of Ibadan, Ibadan, Nigeria; 4 Active Voices, Abuja, Nigeria; 5 Institute of Child Health, University of Ibadan, Ibadan, Nigeria

**Keywords:** PPMVs/drug shops, Private sector, Injectable contraceptives, Nigeria, Job aids, Task sharing

## Abstract

**Background:**

To increase access to voluntary family planning (FP) services, Nigerian policymakers are debating how to task share injectable contraceptive services to drug shop owners known as Patent and Proprietary Medicine Vendors (PPMVs). Task sharing FP services to drug shops is a promising practice, but information is needed on how to ensure high quality FP services. This analysis assesses the effects of job aids on PPMVs’ knowledge of injectable contraceptives 9 months after receiving a standardized training.

**Methods:**

One hundred ninety-four PPMVs were trained on FP counseling and administration of injectable contraceptives in Bauchi, Cross River, Ebonyi and Kaduna states. PPMVs were interviewed before, after, and 9 months after the training. Three variables were used to assess injectable contraceptive knowledge: 1) intramuscular depot-medroxyprogesterone acetate (DMPA-IM) knowledge: a combination of three questions related to device type, injection location, and reinjection frequency; 2) subcutaneous DMPA (DMPA-SC) knowledge: a combination of the same three questions but for DMPA-SC; and 3) knowledge of at least 4 of the 7 common injectable side effects. Three separate adjusted logistic regression models were conducted to determine the factors that influence PPMV knowledge of injectable contraceptives 9 months after the training.

**Results:**

Over half of PPMVs (56%) reported using at least two job aids at 9 months. PPMVs’ knowledge of DMPA-IM, DMPA-SC and side effects were low before the training but increased immediately after. Nine months later, knowledge remained higher than pre-test levels but generally reduced compared to posttest levels. PPMVs who reported using at least two FP job aids were 2.6 (95% CI: 1.4–5.1) times more likely to have DMPA-IM knowledge 9 months after the training compared to those who used one or no job aids, while adjusting for PPMV characteristics. Similar results were observed for knowledge of DMPA-SC (AOR: 2.5; 95% CI: 1.2–4.6) and side effects (AOR: 2.5; 95% CI: 1.3–4.8).

**Conclusion:**

PPMVs who used at least two FP job aids were more likely to correctly answer key injectable contraceptive questions 9 months after training. Incorporating proven job aids into routine trainings is a low-cost strategy that can reinforce knowledge and help PPMVs to retain information.

## Background

Approximately 28% of married (or in-union) women of reproductive age in Nigeria have an unmet need for family planning (FP)- 19% for spacing and 9% for limiting pregnancies [1]. Yet few married women (11%) are using a modern contraceptive method as of 2016 [1]. This is a one percentage point increase in Nigeria’s modern contraceptive prevalence rate from the 2013 National Demographic and Health Survey [2]. Among the 11% of married or in-union women who are using modern contraceptives, 40% use the injectable, 21% use the pill, 13% use the implant, 11% use condoms, and 15% use various other methods [1]. Most (60%) modern contraceptive users obtain their method from the private sector compared to 29% who seek services from the public sector [2]. Of both public and private sources, private-sector Patent and Proprietary Medicine Vendors (PPMVs) are the most popular with 38% of modern contraceptive users obtaining their last method from a PPMV: 52% of pill users, 13% of injectable users and 38% of condom users [2].

Nigeria’s Federal Ministry of Health (FMoH) is dedicated to improving access to voluntary FP services for all women and couples across the country by partnering with the private sector. During the 2012 Family Planning Summit, the FMoH committed to working with the private sector and global partners to reach a modern contraceptive prevalence of 27% by 2020 [3]. In 2017, the FMoH further committed to “expanding the implementation of its task-shifting policy to include PPMVs and community resource persons to improve access to FP services in difficult-to-reach areas and among disadvantaged populations” [3]. Since some women, especially young and unmarried women [4], already seek contraception from PPMVs [1, 2], the FMoH is exploring task sharing injectable contraceptive services to PPMVs as a way to increase access to contraceptive services.

The World Health Organization defines task sharing as the delegation or distribution of tasks or services among workforce teams, and where appropriate, tasks are shared from highly skilled health workers to healthcare workers with fewer qualifications [5]. While task sharing the provision of oral and injectable contraceptives to public-sector community health workers has been proven safe [6, 7] and has become a common strategy to reach women in underserved areas, many countries restrict private sector drug shop owners and pharmacists from providing these services even though drug shop owners generally have the same education levels as community health workers while pharmacists typically have attained higher qualifications [8].

Task sharing the provision of FP services to drug shops and pharmacies has been identified as a promising high-impact practice to expand access to contraceptive services [9]. Studies in Uganda [10] and Bangladesh [9] have also shown that private-sector drug shops can safely administer injectable contraceptives and clients find these services acceptable. Based on these results, Uganda has begun task sharing injectable contraceptive services to drug shops [11]. PPMVs in Nigeria are comparable to private-sector drug shop owners in other countries. They are licensed by State Ministries of Health to sell over-the-counter, pre-packaged medications [12] but are prohibited from prescribing medications or performing invasive procedures such as in injections [13]. There are no educational or training requirements for PPMV licensure [14] and the perception among some in Nigeria is that PPMVs have limited education because there is no educational requirement. Many studies have found, however, that the majority of PPMVs have attained a secondary education or higher [15–18].

PPMVs are an important source of care for many Nigerians. In a systematic review of PPMVs in health care provision, Beyeler et al. [17] found that in addition to FP, PPMVs provide care for up to 55% of under-five child illnesses. PPMVs are often the first point of care for many Nigerians because they are located throughout rural and urban parts of the country with approximately 24 shops per 100,000 people [14]. In addition to their prevalence, they are popular because of their: (a) consistent drug stocks; (b) convenient hours of operation; (c) personable interactions; (d) lack of separate fees for consultations; and (e) anonymous care [19–21].

Although PPMVs are a popular source for FP, previous studies in Nigeria have found mixed results related to their ability to provide contraceptive services. Two studies provided a snapshot of PPMVs’ knowledge and provision of contraceptive services. Ajuwon et al. [16] found that among PPMVs who provide injectables to their clients, their knowledge of injectable contraceptives was low, but clients were satisfied with the injectable services received. Another study showed that PPMVs’ knowledge of oral contraceptives was also low and that many PPMVs did not adhere to FP regulations like requiring a prescription before dispensing oral contraceptives [22]. Studies that have assessed drug shop owners’ knowledge and service provision after training, however, show more promising results. A study in six states in Nigeria found that PPMVs had higher knowledge of injectable contraceptives after receiving a training [15]. Drug shop owners in Uganda also demonstrated increased knowledge after receiving a training and clients reported receiving quality services [23]. These results suggest that PPMVs require standardized training and supportive environments that enable them to consistently offer high quality FP services after being trained in FP services.

In addition to training and ongoing monitoring, another strategy that could support PPMVs in providing FP services is to train them in the use of proven FP job aids. Job aids provide healthcare workers with procedural, informational or decisional “need-to-know” information in a simplified way [24]. Job aids include posters, cue cards, and algorithms, among other tools [24]. They help address challenges to service delivery like provider forgetfulness of highly technical information or procedures involving multiple steps, and in some situations increase efficiency when time is constrained [24]. In Uganda and the Democratic Republic of Congo, Tumlinson et al. [25] found that about half of the providers surveyed reported using FP checklists between 7 and 24 months after training. Those who used these job aids found them to be “very useful.” A study in South Africa found that a FP job aid (a reinjection screening checklist) assisted providers in excluding pregnancy for late-arriving depot-medroxyprogesterone acetate (DMPA) clients and thereby addressed unintentional discontinuation among those clients [26]. They found no difference in clients’ timeliness for reinjections, however. To contribute to the current policy discussions in Nigeria and in similar settings, this analysis assesses the effects of job aids on PPMVs’ knowledge of injectable contraceptives 9 months after receiving a standardized training.

## Methods

### Data source

Data for this analysis come from a larger implementation science study conducted between 2015 and 2018 that looked at the feasibility and acceptability of PPMVs’ provision of progestin-only injectable contraceptives [19]. This analysis uses data collected from PPMVs in four Nigerian states - Bauchi, Cross River, Ebonyi and Kaduna – at three time points. The regulatory body for PPMVs in Nigeria, the Pharmacy Council of Nigeria (PCN), recruited PPMVs for the study based on the following criteria: (a) licensure with PCN; (b) interest and willingness to participate; (c) ability to read and write in English; and (d) commitment to attend all the training sessions.

### Intervention

Between May and June of 2017, 229 PPMVs were trained in FP counseling and the provision of progestin-only injectable contraceptives (counseling, sale, referral and administration). The training lasted 5 days and was facilitated by two FP trainers certified in Nigeria. The curriculum was based on previously used materials developed by PATH [27] and FHI 360 [28] and covered the following topics: (a) FP counseling; (b) injectable client screening and counseling; (c) intramuscular (DMPA-IM) and sub-cutaneous (DMPA-SC) administration of injectable contraceptives (including the re-injection grace period); (d) commodity storage; (e) sharps disposal; (f) infection prevention practices; and (g) pharmacovigilance. Each PPMV received a copy of the curriculum at the beginning of the training.

During the training, PPMVs were given three job aids: (a) the Contraceptive Medical Eligibility Criteria (MEC) wheel; (b) Balanced Counseling Strategy Plus (BCS+) cards; and (c) a DMPA screening check list. The MEC wheel provides information on which contraceptive methods are safe for women based on their health characteristics [29]. BCS+ cards assist health care workers in providing clients with targeted and quality FP counseling [30]. The DMPA screening checklist assists providers to screen injectable contraceptive clients based on their medical eligibility [28].

At the end of the training, PPMVs were required to demonstrate competency in DMPA-IM and DMPA-SC administration on dummy models before continuing in the study. The trainers used a standard observation checklist to determine competency. Four PPMVs were excluded from the study because they were unable to demonstrate competency. After the training, a monitoring team comprised of the research team, trainers, and federal, state, and local Ministry of Health representatives visited PPMVs to identify knowledge gaps and challenges, and to offer feedback to PPMVs. PPMV-client interactions were not observed during these visits and PPMVs were informed when the monitoring visit would take place.

### Data collection

Eight data collectors administered three interviews to PPMVs- a pre-test interview immediately before the training, a posttest interview immediately after the training, and a follow-up interview 9 months after the training. The data collectors were trained in research ethics, the study’s design and the questionnaires approximately 2 weeks before PPMVs were trained. Informed consent was received from PPMVs before the pre-test interview, and before the start of the 9-month interview. All three tools included identical knowledge questions on injectable contraceptives (e.g. frequency and administration location, counseling on side effects, and eligibility criteria). The pre-test interview also included questions on respondent characteristics and their experience providing injectable contraceptives. The 9-month interview included questions on PPMVs’ provision of injectable contraceptives and FP services, and their experience with the intervention (e.g. training, job aids and monitoring visits).

### Dependent variables

Three outcome variables were used to assess PPMV’s knowledge of progestin-only injectable contraceptives: (1) DMPA-IM knowledge; (2) DMPA-SC knowledge; and (3) progestin-only injectable side effect knowledge. The DMPA-IM variable was created by combining three knowledge questions: (a) the type of device used to administer DMPA-IM; (b) where DMPA-IM can be administered on the body; and (c) the reinjection frequency. A dichotomized variable was created and coded as 1 if the PPMV answered all aspects of each question correctly, and 0 if the PPMV answered any part of the three questions incorrectly. The DMPA-SC knowledge variable was based on the same three questions listed above but were specific to DMPA-SC. A similar dichotomized variable was created and coded as 1 if the PPMV answered all three questions correctly and 0 if they answered any question incorrectly.

The side effect variable was based on PPMVs unpromoted response to the question “what are the common side effects of progestin-only injectable contraceptives.” The seven possible responses include: change in menstruation, headaches, dizziness, weight gain, mild skin irritation, decrease in sex drive, and delayed return to fertility. A dichotomized variable was created and coded as 1 if the PPMV named at least 4 of the 7 common side effects and 0 if the PPMV named less than 4 of these side effects. Four was used as the cut-off based on the distribution of side effects PPMVs correctly named at the posttest interview.

### Independent variables

The main predictor was PPMVs’ self-reported use of job aids. At the 9-month interview, PPMVs were asked whether they used the jobs aids distributed during the training when providing FP services to their clients. Those who reported using job aids were then asked which job aids they used. The variable was dichotomized and those who reported using none or one of the job aids were coded as 0 and those who reported using two or three job aids were coded as 1. The other main predictor variable was administration of an injectable in the 30 days preceding the survey to determine whether providing injectable contraceptives recently affected PPMVs’ knowledge. The variable for administering an injectable contraceptive in the past 30 days varied by model. Administration of DMPA-IM in the past 30 days to a client was used for the model focused on DMPA-IM and administration of DMPA-SC in the past 30 days to a client was used for the model focused on DMPA-SC. For the model with side effect knowledge as the outcome, administration of any type of progestin-only injectable was used. These variables were coded as 1 if the PPMV reported administering the injectable to at least one client in the past 30 days and 0 if they did not administer an injectable in that timeframe.

Additional covariates that were considered in the analysis include: (a) sex; (b) age; (c) education; (d) marital status; and (e) state where PPMV shop is located. Sex, age, education and marital status were included as co-variates to account for variation due to participant characteristics. State was included to account for any regional differences. Other variables considered due to theoretical importance were previous health facility experience and receiving a monitoring visit by the study team. Previous health facility experience was not included in the model because it was not a statistically significant predictor of any of the outcome variables at the post-test or 9-month interview in the bivariate analyses. Receiving a monitoring visit was also excluded from the models because almost all PPMVs received the 6-month monitoring visit.

Age was dichotomized at the median age of 35 and the variable was coded 1 if the PPMV was 35 or older and 0 if 34 or younger. Education was dichotomized so that those who had at least 2 years of post-secondary education were coded as 1 and those who had completed a primary or secondary education were coded as 0 (all the participants had a least a primary education). Marital status was dichotomized, and the variable was coded as 1 for those who were currently married and 0 for those who were single, divorced or widowed. State was a categorical variable with a category for each of the four participating states.

### Data analysis

The sample was restricted to PPMVs who completed all three interviews. Of the 225 PPMVs enrolled in the study, 31 PPMVs were unavailable at the time of the 9-month interview, bringing the analytical sample to 194. Descriptive statistics were calculated for PPMV characteristics. Pearson chi-square tests were used to assess trends in knowledge over time. Significance was determined using a probability value of 0.05 or less. Unadjusted odds ratios were first calculated to assess whether certain PPMV characteristics predicted the likelihood of DMPA-IM, DMPA-SC, and side effect knowledge. To determine factors associated with injectable contraceptive knowledge at the 9-month interview, one multivariate logistic regression model was conducted for each of the three outcome variables.

## Results

### Patent and Proprietary Medicine Vendor characteristics

Table 1 presents the background characteristics of PPMVs collected at the pre-test interview. The majority of PPMVs were male (76%) and were married (72%). More than half (55%) were 35 or older and 57% had completed more than a secondary education. Few PPMVs (6%) had completed only primary education (data not shown). Twenty-nine percent reported ever working in a health facility and 44% reported they had administered an injectable contraceptive to a client in their shop before the training.

**Table 1:** PPMV characteristics at the pre-test interview (*N* = 194)

	Percent	Number
Sex		
Male	75.8	147
Female	24.3	47
Age		
17–34	45.4	88
35+	54.6	106
Marital status		
Single/separated/divorced/widowed	27.8	54
Married/in-union	72.2	100
Highest education level achieved		
Primary-secondary education	43.3	84
Two+ years of post-secondary education	56.7	110
Has ever worked in a health facility		
Yes	28.9	56
No	71.1	138
Had ever administered an injectable contraceptive to client in their shop		
Yes	43.8	85
No	56.2	109
State		
Bauchi	27.3	53
Cross River	23.2	45
Ebonyi	26.8	52
Kaduna	22.7	44

Table 2 presents injectable contraceptive services provided and use of job aids at the 9-month interview. Sixty-seven percent of PPMVs reported they had administered an injectable contraceptive to at least one client in the 30 days preceding the interview. More PPMVs reported administering DMPA-IM (56%) compared to DMPA-SC (23%). The mean number of injectable contraceptives administered in the 30 days preceding the 9-month interview was 3 (1.5 for DMPA-IM and 0.5 for DMPA-SC, data not shown).

**Table 2:** Reported services provided by PPMVs at the 9-month interview (*N* = 194)

	Percent
Had administered any progestin-only injectable to a client in the past 30 days	67.0
Had administered DMPA-IM to a client in the previous 30 days	55.7
Had administered DMPA-SC to a client in the previous 30 days	23.2
Reported using job aids with at least some of FP clients^a^	
0 job aids	13.4
1 job aid	29.4
2 job aids	29.3
3 job aids	26.3

^a^2 missing observations

PPMVs were asked whether they used jobs aids while providing FP services during the 9 months since the training. Most PPMVs (87%) reported using at least one of the three FP job aids provided as part of the intervention: 27% of PPMVs reported using all three, 30% used two, 29% used one and 13% used none of the job aids. Of the three job aids provided, 68% of PPMVs reported using the MEC wheel, 61% used the BCS+ counseling cards, and 40% reported using the DMPA screening checklist (data not shown).

### PPMV trends in knowledge over time

**Fig. 1:**
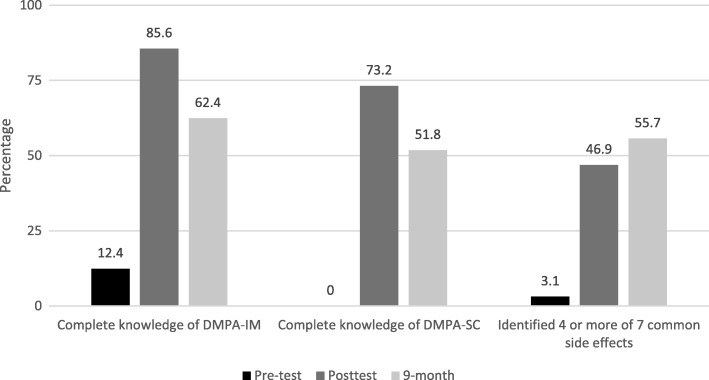
PPMV knowledge of DMPA-IM and DMPA-SC characteristics, and of 4 or more side effects at pre-test, posttest and 9-month follow-up interviews (*N* = 194)

Figure 1 presents trends in PPMVs’ knowledge on the three knowledge variables from the pre-test, posttest and 9-month interviews. DMPA-IM and DMPA-SC knowledge (administration device, where the injectable can be administered on the body, and reinjection frequency) were low at the pre-test interview and increased significantly by the posttest interview. For example, the proportion of PPMVs who correctly identified all three questions related to DMPA-IM increased from 12% at pre-test to 86% at the posttest interview (*p*-value < 0.01). Between the posttest and 9-month interview, however, the proportion of PPMVs who had DMPA-IM knowledge decreased from 86 to 62% (*p*-value < 0.01). A similar trend was observed for DMPA-SC knowledge. DMPA-IM and DMPA-SC knowledge at the 9-month interview was still significantly higher than knowledge at the pre-test interview (*p*-value < 0.01).

PPMV’s knowledge of side effects increased from the pre-test to posttest interview and continued to increase after the training. The proportion of PPMVs who could name at least 4 of the 7 common side effects increased from 3% at the pre-test interview to 47% at the posttest interview (*p*-value < 0.01), and then to 56% at the 9-month interview but this difference was not statistically significant from the posttest interview (*p*-value < 0.05).

### Effect of reported use of job aids on DMPA-IM and DMPA-SC knowledge

Two logistic regression models were conducted to assess factors that predicted the likelihood of DMPA-IM and DMPA-SC knowledge at the 9-month interview. Table 3 shows the unadjusted and adjusted odds ratios of DMPA-IM knowledge. The odds of PPMVs accurately answering all three DMPA-IM questions was 2.8 times more likely among those who used at least two jobs aids compared to those who used one or none (OR 2.8 *p*-value < 0.01; 95% CI 1.5–5.2). When adjusting for sex, age, education, marital status, state and administration of DMPA-IM in the past 30 days, the odds ratio went down slightly to 2.7 but remained significant (AOR 2.7 *p*-value < 0.01 95% CI 1.4–5.1).

**Table 3:** Unadjusted and adjusted odds ratios of DMPA-IM knowledge at 9-month interview (*N* = 194)

	Unadjusted odds		Adjusted odds^a^	
	Odds ratio	95% CI	Odds ratio	95% CI
Number of job aids used when providing FP counseling^a^				
Used 0 or 1	ref	–	–	–
Used 2 or 3	2.83**	(1.54–5.17)	2.65**	(1.37–5.12)
Sex				
Female	ref	–	–	–
Male	0.86	(0.44–1.66)	0.91	(0.42–1.96)
Age				
34 or younger	ref.	–	–	–
35 or older	1.18	(0.66–2.12)	1.16	(0.58–2.33)
Had 2 or more years of higher education				
No	ref	–	–	–
Yes	0.72	(0.40–1.31)	0.90	(0.42–1.92)
Marital status				
Not married	ref	–	–	–
Currently married	1.08	(0.56–2.05)	0.99	(0.45–2.18)
Injected DMPA-IM in the past 30 days				
No	ref	–	–	–
Yes	1.26	(0.70–2.26)	1.02	(0.54–1.96)
State				
Bauchi	ref	–	–	–
Cross River	1.75	(0.66–3.24)	1.24	(0.45–3.45)
Ebonyi	1.46	(0.34–1.74)	1.20	(0.48–3.00)
Kaduna	0.78	(0.82–2.43)	0.76	(0.30–1.90)

** *p*-value < 0.01

^a^2 missing observations

Table 4 shows the unadjusted and adjusted odds ratios for DMPA-SC knowledge. These results are similar to the results in the DMPA-IM models. Among those PPMVs who reported using two or three job aids, they were 2.5 times (OR 2.5 *p*-value < 0.01 95% CI 1.4–4.5) more likely to answer the three questions about DMPA-SC correctly 9-months after the training. When adjusting for covariates, the odds ratio for remained the same (AOR = 2.5; *p*-value < 0.01 95% CI 1.2–4.6).

**Table 4:** Unadjusted and adjusted odds ratios of DMPA-SC knowledge at 9-month interview (*N* = 194)

	Unadjusted odds		Adjusted odds^a^	
	Odds ratio	95% CI	Odds ratio	95% CI
Number of job aids used when providing FP counseling^a^				
Used 0 or 1	ref	–	–	–
Used 2 or 3	2.48**	(1.37–4.45)	2.45**	(1.23–4.62)
Sex				
Female	ref	–	–	–
Male	1.92	(0.97–3.77)	1.82	(0.84–3.95)
Age				
34 or younger	ref	–	–	–
35 or older	0.78	(0.44–1.38)	0.94	(0.47–1.87)
Had 2 or more years of higher education				
No	ref	–	–	–
Yes	1.14	(0.64–2.01)	1.45	(0.68–3.08)
Marital status				
Not married	ref	–	–	–
Currently married	0.69	(0.36–1.31)	0.62	(0.27–1.39)
Injected DMPA-SC in the past 30 days				
No	ref	–	–	–
Yes	1.74	(0.88–3.44)	1.84	(0.85–3.97)
State				
Bauchi	ref	–	–	–
Cross River	2.36*	(1.04–5.35)	1.64	(0.59–4.50)
Ebonyi	1.16	(0.54–2.51)	1.19	(0.48–2.97)
Kaduna	1.57	(0.70–3.50)	1.29	(0.52–3.24)

* *p*-value < 0.05; ** *p*-value < 0.01

^a^2 missing observations

### Effect of reported job aids use on knowledge of 4 or more common side effects

Table 5 shows the unadjusted and adjusted odds ratios of PPMV’s knowledge of common side effects of injectables contraceptives. PPMVs who used two or three job aids were 2.1 times more likely to know 4 or more common side effects (OR 2.1 *p*-value < 0.01 95% CI 1.2–3.8). When adjusting for covariates, use of two or three jobs aids remained significant (AOR = 2.5; *p*-value < 0.01 95% CI 1.3–4.8). PPMVs with more than a secondary education were about 2 times more likely than those with a primary or secondary education in the unadjusted model (OR = 1.8; *p*-value < 0.05 95% CI 1.0–3.2). The effect of education, however, was attenuated in the adjusted model.

**Table 5:** Unadjusted and adjusted odds ratios for knowledge of 4 or more side effects at 9-month interview (*N* = 194)

	Unadjusted odds		Adjusted odds^a^	
	Odds ratio	95% CI	Odds ratio	95% CI
Number of job aids used when providing FP counseling^a^				
Used 0 or 1	ref	–	–	–
Used 2 or 3	2.14**	(1.19–3.84)	2.50**	(1.29–4.84)
Sex				
Female	ref	–	–	–
Male	1.23	(0.63–2.40)	1.31	(0.61–2.80)
Age				
34 or younger	ref	–	–	–
35 or older	0.84	(0.48–1.49)	0.84	(0.43–1.65)
Had 2 or more years of higher education				
No	ref	–	–	–
Yes	1.78*	(1.00–3.17)	1.32	(0.65–2.73)
Marital status				
Not married	ref	–	–	–
Currently married	1.12	(0.59–2.10)	1.05	(0.49–2.28)
Injected any injectable contraceptive in the past 30 days				
No	ref	–	–	–
Yes	1.28	(0.70–2.34)	0.87	(0.43–1.75)
State				
Bauchi	ref	–	–	–
Cross River	0.75	(0.34–1.68)	0.71	(0.27–1.89)
Ebonyi	0.56	(0.26–1.22)	0.60	(0.24–1.46)
Kaduna	1.15	(0.50–2.62)	1.17	(0.46–2.96)

* *p*-value < 0.05; ** *p*-value < 0.01

^a^2 missing observations

## Discussion

This study showed that PPMVs who reported using at least two job aids were more likely than those who reported using less than two to have correct knowledge of DMPA-IM, DMPA-SC and injectable contraceptive side effects 9 months after being trained in FP counseling and injectable contraceptive provision. Previous studies primarily focused on the current situation in Nigeria where PPMVs are not routinely trained to provide contraceptive services and found that contraceptive knowledge and adherence to clinical guidelines were low [16, 22]. This study explored knowledge among PPMVs who received training in FP counseling and injectable contraceptives, similar to work done with drug shops in Uganda [23]. Factors that influenced knowledge retention after training were also identified.

Before the training, few PPMVs correctly answered questions related DMPA-IM, DMPA-SC and injectable side effects even though almost half of the PPMVs reported they had previously administered injectable contraceptives to clients. These pre-test results indicating low knowledge of injectable contraceptives are consistent with findings from similar studies in Nigeria and Uganda [16, 23]. This suggests that many PPMVs are providing injectable contraceptive services to meet client demand but without the necessary knowledge to do so.

Knowledge outcomes increased after the training, suggesting that training is the first step in providing PPMVs with the necessary information to provide injectable contraceptives and that PPMVs can learn from a standard curriculum Since the education levels of PPMVs ranged from primary to post-secondary education, basing curricula on previously tested materials geared toward community health volunteers is recommended for future interventions with PPMVs and drug shops owners in similar settings [27, 28].

Nine months after the training, some PPMVs’ knowledge on the key DMPA-IM and DMPA-SC indicators decreased and their knowledge of side effects remained relatively low. While a decrease in knowledge after the training was expected, some PPMVs retained knowledge 9 months afterwards. Results from the multivariate models showed that PPMVs who reported using at least 2 job aids were more likely to answer the key knowledge questions correctly. Job aids, when used, are an effective tool to help health care providers adhere to service protocols and remember key information [23–25]. This study showed that PPMVs can also benefit from using job aids when providing FP services.

A majority of the PPMVs (87%) in this study reported using at least one of the job aids that were provided as part of the intervention. This indicates that PPMVs and drug shop owners are likely to refer to job aids when trained on how to use these tools. The multivariate results showed that PPMVs who used more than one job aid were more likely to retain key information 9 months after the training. This suggests that PPMVs may benefit from using a combination of job aids rather than relying on only one tool.

Among those who reported using job aids, the MEC wheel and BCS+ cards were the most commonly cited (68 and 61% of PPMVs respectively). Fewer (40%) reported using the DMPA screening checklist which may be because the DMPA screening checklist covers similar information included in the MEC wheel. However, considering that about a third of PPMVs reported using all three, future interventions should offer newly trained cadres more than one job aid so that providers can choose between tools depending on the situation, the client, or their preference. As with the curriculum, providing job aids that have been previously tested is suggested in similar interventions with PPMVs or with drug shop owners in other settings. Future studies should explore whether job aids have a positive effect on the quality of care received by clients and what factors influence PPMV use of jobs such as type of client, internalization of information, or other shop influences (e.g. number of people present).

Provision of an injectable method in the past 30 days was not found to be a statistically significant predictor of knowledge for any of the three outcomes and therefore administering an injection within 1 month is not sufficient to maintain knowledge among PPMVs or other newly trained cadres. Additional research is needed on whether higher client load or repeated practice results in better knowledge outcomes after training. Finally, a majority of the PPMVs were monitored one and 6 months after the training. Despite this support, many PPMVs had injectable contraceptive knowledge 9 months after the training. Further research is needed on the frequency of supportive supervision and appropriate supervision tools required for PPMVs to provide injectable contraceptives at scale.

### Limitations

The variable “use of job aids” was self-reported rather than observed. While there is a chance that PPMVs over-reported their use of job aids during the 9-month interview, there was no direct benefit to PPMVs for using job aids as part of the intervention. This reduced the chance for any significant overreporting.

Client-PPMV observations were not conducted because this study did not include a demand generation component. Without sufficient demand, an observational component would have required a significant amount of time and resources. For this reason, knowledge outcomes were used instead of behavioral ones. Although knowledge is not a proxy for behavior, based on Issenberg’s 2005 elements for analyzing professional competence, knowledge is the first domain of competence and therefore required before performance in control or real-life settings [31].

Of the 225 PPMVs trained only 194 were available for their 9-month interview. Pearson chi-square tests were used to determine whether the sample used for this analysis (*n* = 194) had similar background characteristics to the overall sample from the study (*n* = 225). The results of this sensitivity analysis suggested that the two groups were not statistically different (*p*-value < 0.05).

## Conclusion

Results from this and previous studies have shown that drug shop operators, such as PPMVs, are already providing injectable contraceptives yet they require support to have the necessary knowledge and skills to provide injectable contraceptives properly. Training is a necessary first step but is not enough to ensure knowledge over time. Job aids are a low-cost way to assist PPMVs, and other newly trained FP providers, in remembering key details, especially long lists of information such as side effects. Monitoring and supervision are also important aspects to ensure safe and quality services from PPMVs. With comprehensive support, PPMVs have the potential to be a viable source for injectable contraceptive services for women in Nigeria. The results from this study contribute to the existing literature showing that task sharing FP services to drug shops is a feasible practice to help governments expand access to voluntary FP services that meet clients’ needs.

## Data Availability

The dataset analyzed during the current study will be available at the USAID Development Data Library in January 2021, https://www.usaid.gov/data. They will also be available from the corresponding author on reasonable request.

## Abbreviations

BCS: Balanced Counseling Strategy Plus
DMPA: Depot-medroxyprogesterone acetate
FMOH: Federal Ministry of Health
IM: Intramuscular
MEC: Medical Eligibility Criteria
PCN: Pharmacy Council of Nigeria
PPMV: Patent and Proprietary Medicine Vendor
SC: Sub-cutaneous
